# Micro (mRNA) molecules could pack a big punch in the fight against neuromuscular disease

**DOI:** 10.1113/JP280872

**Published:** 2020-10-26

**Authors:** Emma Rybalka, Cara A. Timpani

**Affiliations:** ^1^ Institute for Health and Sport (IHeS) Victoria University Melbourne Victoria 8001 Australia; ^2^ Australian Institute for Musculoskeletal Science (AIMSS) St Albans Victoria 3021 Australia

**Keywords:** duchenne muscular dystrophy, dystrophin, micro RNAs, muscle regeneration, satellite cells, skeletal muscle

In the fatal neuromuscular disease, Duchenne muscular dystrophy (DMD), exhaustion of the muscle stem (satellite) cell pool drives muscle wasting through mismatched degeneration and regeneration processes (Forcina *et al*. 2019). For this reason, the development of regenerative myoblast transfer therapeutics has been pursued for more than two decades, but to no avail. Skeletal muscle is a dynamically plastic organ capable of responding and adapting to mechanical and biochemical injury so as to match the physicality and functionality of muscles to the external demands placed upon them. In this way, humans can grow bigger and stronger muscles or, conversely, leaner yet more functionally durable muscles to promote organismal survival according to environmental cues. As such, skeletal muscle is in a constant state of ‘turnover’ facilitated by satellite cell proliferation, differentiation and self‐renewal (Forcina *et al*. 2019). These processes are highly amenable to amplification by a variety of factors when more muscle tissue is required, i.e. during growth or muscle degeneration/injury. Chronic pathologies such as DMD present a unique internal landscape which challenges muscle plasticity (Dumont & Rudnicki, 2016) and, consequently, human survival.

Satellite stem cells reside in a niche between the sarcolemma and basal lamina (Forcina *et al*. 2019). They are particularly responsive to inflammatory and muscle‐specific cytokines, which are released during stress, damage and inflammation (Forcina *et al*. 2019). Their activity is also highly regulated by growth factors, local and distant non‐muscle cells (such as fibro‐adipocytes, immune cells etc.), proteins associated with the dystrophin‐associated glycoprotein complex (DGC), micro and long non‐encoding RNAs, and telomeric activity (Forcina *et al*. 2019). When quiescent satellite cells are activated to re‐enter the cell cycle, two daughter cells are produced which have one of two potential fates. Asymmetric division produces one committed muscle progenitor and one stem cell to facilitate both muscle repair and self‐renewal of the satellite pool (Dumont & Rudnicki, 2016). In contrast, symmetric division enables rapid expansion of the satellite cell pool by producing two stem cells (Dumont & Rudnicki, 2016). A balance between asymmetric and symmetric division is crucial for enabling rapid muscle repair during acute injury, as well as for maintaining the long‐term regenerative potential of skeletal muscle. Just as important is the capacity of satellite cells to flux between activation/proliferation and periods of quiescence (Dumont & Rudnicki, 2016). This is because with each cell cycle, telomeric DNA is shortened and the capacity for satellite cell proliferation is proportionately diminished unto exhaustion (Forcina *et al*. 2019). Dystrophic satellite cells, which carry loss‐of‐function mutation(s) in the dystrophin gene, manifest stark differences in function compared to healthy satellite cells (Dumont & Rudnicki, 2016). Dystrophin protein, as well as other DGC proteins, is integral for maintaining asymmetric satellite cell division and without it, muscle regenerative capacity is compromised (Dumont & Rudnicki, 2016).

Micro‐RNAs (miR) are short, non‐encoding RNAs that have emerged as important post‐transcriptional regulators of many genes including those that control satellite cell activity, muscle growth and regeneration (Forcina *et al*. 2019). In this issue of *The Journal of Physiology*, Taetzsch *et al*. (2021) describe for the first time the temporal changes in muscle‐specific miR‐133b throughout the pathogenesis of murine (*mdx*) DMD and the effect of miR‐133b deletion on the *mdx* phenotype. Although it is the ‘gold‐standard’ animal model of DMD because of its genetic homology to human DMD, the *mdx* mouse has a peculiar phenotype making translational research challenging. Many studies fail to recognise that *mdx* mice enter a stabilised ‘remission’ phase from ∼5 postnatal weeks of age, following an intense muscle damage, inflammation and regeneration phase lasting from ∼3 to 5 postnatal weeks of age. Taetzsch *et al*. address this aspect by investigating multiple time points and importantly show that miRNA‐133b is robustly expressed in *mdx* muscles, and significantly more so in the severe compared to the remission phase of *mdx* MD (Taetzsch *et al*. 2021). Genetic knockout of miR‐133b had little effect on wild‐type muscles, yet it exacerbated MD in *mdx* mice, suggesting that miR‐133b attenuates the severity of DMD‐associated myopathy (Taetzsch *et al*. 2021). Taetzsch *et al*.’s data highlight that miR‐133b is crucial for mediating the molecular response to damage which facilitates repair and regeneration of skeletal muscle and could thus be targeted through genetic or pharmacological intervention as a treatment for DMD.

While the mechanisms governing miR‐133b's influence on DMD were not extensively investigated, an interesting revelation was that pathways involving STAT3 and TGF‐β/Smad were upregulated while pathways governing fat metabolism were downregulated in *mdx/miR‐133b^−/−^* muscles (Taetzsch *et al*. 2021). Paradoxically, STAT3 signalling is associated with both hypertrophy and atrophy of muscles through controlling satellite cell fate – presumably by manipulating the balance between proliferation and differentiation (Guadagnin *et al*. 2018). When STAT3 inhibitors are administered to *mdx* mice, muscle repair and regeneration is sustained through expansion of the satellite cell pool but when STAT3 is depleted, the satellite pool gradually diminishes and MD is exacerbated (reviewed in Guadagnin *et al*. 2018). miR‐133b appears to be a strong regulator of STAT signalling to enhance muscle regeneration, and/or of other similarly functioning satellite regulators such as myostatin. Myostatin is a growth factor of the TGF‐β superfamily and induces Smad‐dependent repression of postnatal muscle growth (Guadagnin *et al*. 2018). However, very different roles are purported for myostatin during embryonic myogenesis, where it controls satellite cell fate in a similar fashion to STAT3 (Guadagnin *et al*. 2018). Other miRNAs have established regulatory function over myostatin and so too might miR‐133b, either directly or through interplay with other miRs. Altering metabolic substrate utilisation could be another therapeutic mechanism of miR‐133b, since dystrophin‐deficient muscles have well‐documented mitochondrial and other metabolic deficits, which, when therapeutically targeted, can attenuate MD (Timpani *et al*. 2020). Although the precise mechanisms require unravelling, Taetzsch *et al*. highlight miR‐133b as a potential ‘micro’ therapeutic target against the severity and progression of DMD.

## Additional information

### Competing interests

There are no competing interests to declare.

### Author contributions

Both authors contributed equally. Both authors have approved the final version of the manuscript and agree to be accountable for all aspects of the work. All persons designated as authors qualify for authorship, and all those who qualify for authorship are listed.

### Funding

There was no specific funding attributable to this work.
